# The Murid Herpesvirus-4 gH/gL Binds to Glycosaminoglycans

**DOI:** 10.1371/journal.pone.0001669

**Published:** 2008-02-27

**Authors:** Laurent Gillet, Susanna Colaco, Philip G. Stevenson

**Affiliations:** Division of Virology, Department of Pathology, University of Cambridge, Cambridge, United Kingdom; Institut Pasteur Korea, Republic of Korea

## Abstract

The first contact a virus makes with cells is an important determinant of its tropism. Murid Herpesvirus-4 (MuHV-4) is highly dependent on glycosaminoglycans (GAGs) for cell binding. Its first contact is therefore likely to involve a GAG-binding virion glycoprotein. We have previously identified two such proteins, gp70 and gp150. Gp70 binds strongly to GAGs. However, deleting it makes little difference to MuHV-4 cell binding or GAG-dependence. Deleting gp150, by contrast, frees MuHV-4 from GAG dependence. This implies that GAGs normally displace gp150 to allow GAG-independent cell binding. But the gp150 GAG interaction is weak, and so would seem unlikely to make an effective first contact. Since neither gp70 nor gp150 matches the expected profile of a first contact glycoprotein, our understanding of MuHV-4 GAG interactions must be incomplete. Here we relate the seemingly disconnected gp70 and gp150 GAG interactions by showing that the MuHV-4 gH/gL also binds to GAGs. gH/gL-blocking and gp70-blocking antibodies individually had little effect on cell binding, but together were strongly inhibitory. Thus, there was redundancy in GAG binding between gp70 and gH/gL. Gp150-deficient MuHV-4 largely resisted blocks to gp70 and gH/gL binding, consistent with its GAG independence. The failure of wild-type MuHV-4 to do the same argues that gp150 is normally engaged only down-stream of gp70 or gH/gL. MuHV-4 GAG dependence is consequently two-fold: gp70 or gH/gL binding provides virions with a vital first foothold, and gp150 is then engaged to reveal GAG-independent binding.

## Introduction

Many herpesviruses use glycosaminoglycans (GAGs) for their first cell contact. Murid Herpesvirus-4 (MuHV-4) is highly dependent on GAGs for cell binding and infection [1]. Its ORF4 encodes a strong GAG-binding virion glycoprotein, gp70 [2], which is homologous to the Kaposi's Sarcoma-associated Herpesvirus ORF4 gene product [3], and analogous to the Herpes Simplex virus gC [4]: all are complement control proteins that also bind to GAGs [5]. But if a need for GAG binding by gp70 explained MuHV-4 GAG-dependence, then gp70-deficient MuHV-4 should bind poorly to GAG^+^ cells, much as the wild-type binds poorly to GAG^−^ cells [6]. Instead, gp70-deficient MuHV-4 shows little deficit in binding or infection, and is if anything better inhibited by soluble heparin than the wild-type [2]. Also, much of the gp70 on MuHV-4 virions is post-translationally cleaved to release its GAG-binding domain [2]. These data suggest that gp70 does not provide a particularly important first contact. It might be more important for saturating the GAGs on productively infected cells to promote virion release, or for absorbing soluble GAGs in infectious foci to stop them inhibiting spread. Alternatively, its GAG binding might be subservient to complement evasion [7].

While the contribution gp70 makes to MuHV-4 GAG-dependence is unclear, there is good evidence for the MuHV-4 gp150 being important [1]. Thus, gp150-specific monoclonal antibodies (mAbs) increase MuHV-4 infection of GAG-deficient cells [8] and gp150^−^ mutants are almost completely GAG-independent: they infect GAG^+^ cells normally, infect GAG^−^ cells much better than the wild-type does, and largely resist inhibition by soluble heparin. Interestingly, the gp150 homolog of Epstein-Barr virus (EBV), gp350, has a similar inhibitory role in making EBV infection CD21-dependent [9], [10]. But unlike the strong CD21 binding of gp350 [11], recombinant gp150 binds to GAGs at best weakly [2]. This argues against gp150 being important for the initial capture of virions onto cell surfaces. Also, gp150 knockouts generally bind better to cells rather than worse. Thus, gp150 is not so much a cell binding protein as a GAG-sensitive switch, constitutively inhibiting MuHV-4 infection until GAGs are engaged.

The combination of an abundantly secreted protein that binds GAGs strongly (gp70) and a regulatory protein that binds GAGs weakly (gp150) makes sense as a virion release mechanism. However, it leaves unanswered how MuHV-4 virions first attach to cells: neither gp70 nor gp150 fits the expected profile of a strong cell binding protein for which the corresponding knockout virus shows a marked binding deficit. MuHV-4 has at least 2 more cell-binding virion glycoproteins that could contribute: gB, which binds to cells as an Fc fusion protein [2]; and gL, since gL-deficient mutants binding less well than the wild-type to adherent cells [6], [12]. Because gB binds to a non-GAG ligand [2], while the MuHV-4 first contact is very likely to involve GAGs, we focussed here on gL.

gL lacks a membrane anchor and resides in the virion envelope by virtue of its association with gH [13]. As with other herpesviruses [14], [15], the presence or absence of gL sets the MuHV-4 gH conformation [12], [13]. Interestingly, glycosyl-phosphatidyl-inositol (GPI)-linked gL still folds gH into its native virion form [13]. gL is therefore likely to lie close to the virion membrane in the mature gH/gL heterodimer. This, and the small size of gL, argue that gL does not bind to cells itself, but rather operates through its effect on gH. In order to understand better how gH/gL, gp70, gp150 and gB work together, and how gL might contribute to virion capture, we expressed recombinant gH/gL and tested it for cell binding. We found that gH/gL, like gp70, binds to GAGs. gH alone did not, explaining why a gL deficiency reduces MuHV-4 binding to adherent cells. GAG binding by gH/gL also explains the redundancy of gp70. In a refined model, we propose that MuHV-4 first engages cells through either gH/gL or gp70 binding GAGs. Gp150 displacement then allows GAG-independent cell binding, probably by gB.

## Results

### GAG binding by recombinant gH/gL

We first attempted to make a soluble MuHV-4 gH/gL heterodimer by co-expressing an Fc fusion of the gH extracellular domain with gL, as done for Herpes simplex virus [16]. This failed. We have previously observed that although gL is bound to gH in virions, the transfected MuHV-4 gL folds GPI-linked gH poorly [13]. gH and gL associate in the endoplasmic reticulum of infected cells, as pulse-labelled, endoglycosidase H-sensitive gH displays gH/gL epitopes [13]. But whereas the Herpes simplex virus gH is retained in the endoplasmic reticulum without gL [14], the MuHV-4 gH is exported [13]. Other MuHV-4 glycoproteins may therefore be needed to keep gH and gL together. Notably, virion gH/gL is normally associated with gB and probably also with gp150 [6].

**Figure 1:**
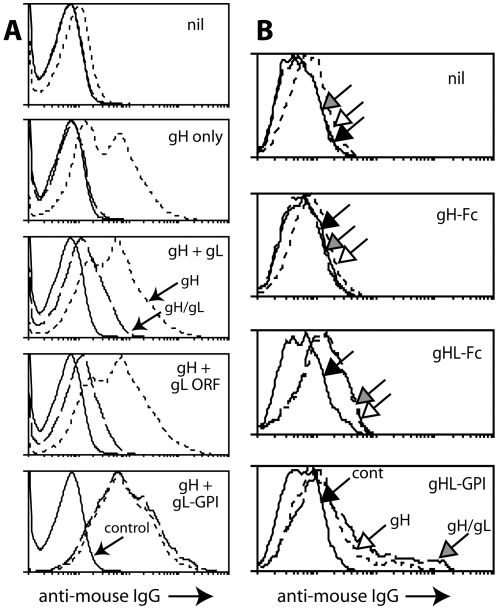
Expression of gH/gL as a single Fc fusion protein. A. 293T cells were transfected with different expression plasmids. 48h later they were trypsinized and analyzed by flow cytometry for expression of gH with mAb 8C1 (short dashes) or gH/gL with mAb 7E5 (long dashes). Control = secondary antibody only (solid lines). “gH+gL ORF” used the full-length genomic ORF47; “gH+gL” used the RACE-mapped gL, which starts at the 5th in-frame ORF47 AUG codon [9]; “gH+gL-GPI” used a GPI-linked form of the RACE-mapped gL. In each case, gH was expressed from the full-length genomic ORF22. nil = untransfected. B. 293T cells were transfected with diferent gH expression plasmids or left untransfected (nil), and 48h later analyzed for gH expression with mAb 8C1 (short dashes, white arrow) and for gH/gL expresion with mAb 7E5 (long dashes, grey arrow), as in A. Solid lines/black arrow = secondary antibody only. gHL-GPI and gHL-Fc are the same fusion protein with either a C-terminal GPI anchor or human IgG_1_-Fc. Each histogram shows 10,000 cells. Both gH-specific and gH/gL-specific staining was significantly increased after gHL-Fc transfection compared with controls (p<0.00001 by Student's t test). Equivalent results were obtained in a repeat experiment.

In contrast to soluble gL, GPI-linked gL folds gH-GPI quite well [13]. This applied also to gH with its native transmembrane domain (Fig. 1A). Some gL immobilization may therefore suffice to stabilize gH/gL. We have previously made a membrane-bound form of gH/gL by fusing GPI-linked gL to the gH extracellular domain [17]. This construct reconstituted the epitopes of 42/42 gH/gL-specific mAbs, all derived from infected mice and specific for conformational epitopes displayed on infected cells (data not shown). We reasoned that a gH-gL fusion protein reproduces the native gH/gL extracellular domain, and so used the same approach to make soluble gH/gL.

**Figure 2:**
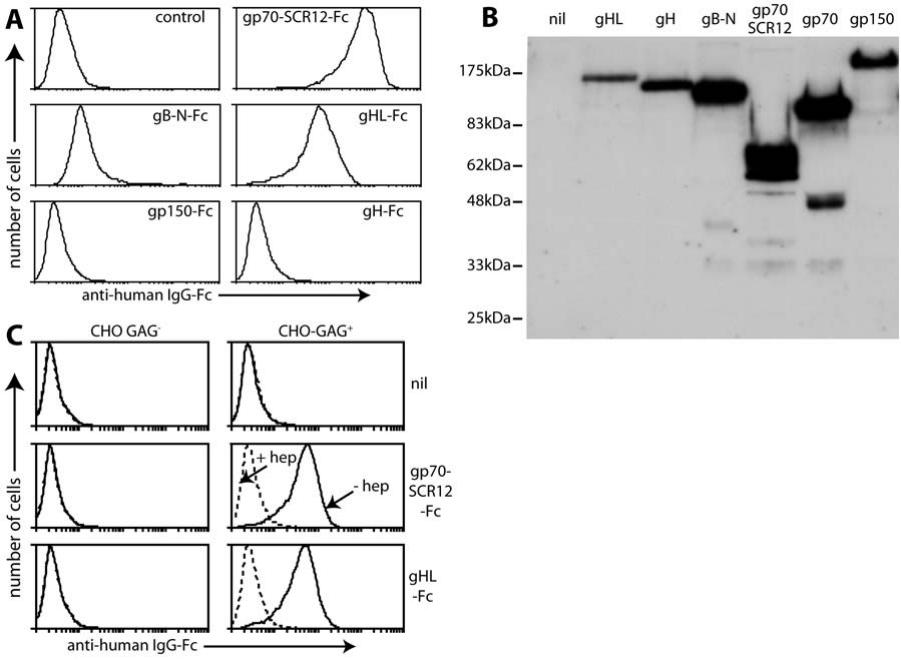
gHL-Fc binds to GAGs. A. Supernatants from 293T cells transfected with expression constructs for different IgG-Fc fusion proteins were used to stain BHK-21 cells. gp70-SCR12 = short consensus repeats 1 and 2 of gp70, which include its heparin-binding site; gB-N = gB N-terminal to its furin cleavage site, which incorporates its cell-binding domain. To retain GAG expression, the cells were plated onto Petri dishes 18h before staining and then detached without trypsinization. Control = untransfected 293T cell supernatant plus secondary antibody. Equivalent data were obtained in 4 repeat experiments. B. The transfected cell supernatants from A were immunoblotted for their common human IgG-Fc domain. nil = untransfected cell supernatant. gp70 = Fc fusion of the full-length protein, which was not used here. C. GAG^+^ CHO cells or the GAG-deficient CHO-745 mutant (GAG^−^) were stained with Fc fusion proteins as indicated, either in the presence (dashed lines) or absence (solid lines) of 300 µg/ml heparin. nil = secondary antibody only. Equivalent data were obtained in 2 repeat experiments.

Flow cytometry established that a gH-gL-Fc fusion (gHL-Fc) reached the plasma membrane of transfected cells and reproduced native gH/gL epitopes (Fig. 1B). gHL-Fc but not gH-Fc recovered from transfected cell supernatants bound to BHK-21 cells (Fig. 2A). Other Fc fusion proteins are shown for comparison. Expression levels for each are shown in Fig. 2B. Gp70-SCR12-Fc incorporates the gp70 GAG-binding domain [2]. gB-N-Fc incorporates the N-terminal MuHV-4 gB furin cleavage product [18], equivalent to domains I and II of the Herpes simplex virus gB [19]. Both bound to BHK-21 cells. The N-terminal third of gp150 fused to GST can bind to GAGs at high dose [2], but neither the same portion of gp150 nor the full-length extracellular domain (shown here) did so when fused to IgG Fc, presumably because gp150 binds to GAGs with very low affinity. Neither gp70-SCR12-Fc nor gHL-Fc bound to GAG-deficient CHO cells, and both had their binding to GAG^+^ CHO cells blocked by pre-incubation with soluble heparin (Fig. 2C). Both therefore depended on GAGs for binding.

### gH/gL precipitation from virion lysates by heparin-agarose

**Figure 3:**
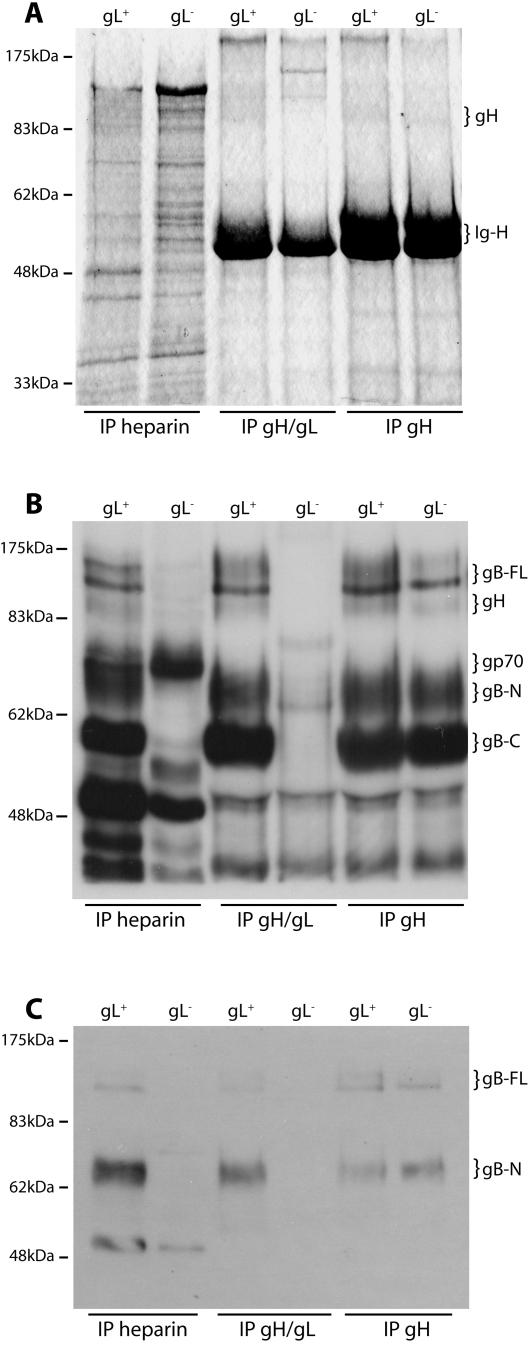
Virion gH/gL binds to GAGs. A. Wild-type (gL^+^) and gL-deficient (gL^−^) virions were lysed in 1% digitonin, then immunoprecipitated with either heparin-agarose or protein A-sepharose plus mAb 7E5 (gH/gL-specific) or 8C1 (which recognizes all forms of gH). The washed precpitates were denatured, resolved by SDS-PAGE and visualized by Coomassie staining. The positions of gH (85 kDa) and IgG heavy chains (Ig-H, 50–55 kDa) are shown. The gH signal was weak but clearly visible by comparison with the gL^−^ virion/gH/gL IP control. The data are from 1 of 2 equivalent experiments. B. The same precipitates as in A were immunoblotted with a MuHV-4-immune rabbit serum, which recognizes (among other virion proteins) gp70, gp150, gB and gH. The MuHV-4 virion glycoproteins are readily distinguished by SDS-PAGE. The positions of gH (85 kDa), gp70, full-length gB (gB-FL, 120 kDa) and the N- and C-terminal gB cleavage products (gB-N, 65 kDa; gB-C, 55 kDa) are shown. The data are from 1 of 2 equivalent experiments. C. The same precipitates were immunoblotted with mAb MG-10C11, which recognizes an epitope in gB-N. The gB-FL bands are faint because most virion gB is cleaved [18]. The identity of the 50 kDa band in the heparin IP lanes is unclear. The only glycoprotein target of MG-10C11 is gB, but it is known to also recognize a 50 kDa virion protein on immunoblots [21].

We previously failed to identify gH/gL by precipitating virion proteins with heparin-agarose [2]. Two factors probably contributed. First, we have no antibody that immunoblots gH, making it harder to detect than gB, gp150 or gp70. Second, we used Triton X-100 virion lysates. Fig. 1 suggests that the MuHV-4 gH and gL do not form a stable heterodimer without other virion proteins. gB may be one, since it associates with gH/gL and hides parts of it from antibody [20]. The gH/gL/gB association is preserved in digitonin but lost in Triton X-100 [6]. Thus, gB dissociation in Triton X-100 may destabilize gH/gL. We have immunoprecipitated gH/gL from Triton X-100 virion lysates with gH/gL-specific mAbs [12]. But mAb binding would provide considerable extra stability; heparin-agarose binding may not. We therefore tested heparin-agarose for gH/gL precipitation using digitonin virion lysates (Fig. 3).

Coomassie staining (Fig. 3A) showed a weak 85 kDa band in gH-specific immunoprecipitates, which corresponds to gH [13]. This band was not obvious in heparin-agarose precipitates due to background bands at the same molecular weight. We therefore immunoblotted the precipitates with an MuHV-4-immune rabbit serum, which (among other virion glycoproteins) recognizes gH [13] (Fig. 3B). This identified an 85 kDa band precipitated by heparin-agarose from gL^+^ but not gL^−^ lysates. (The faint background band in the gL^−^ lane has a slightly lower molecular weight.) The same band was immunoprecipitated from gL^+^ and gL^−^ lysates by a gH-specific mAb and only from gL^+^ virion lysates by a gH/gL-specific mAb, confirming that it was gH (Fig. 3B). The 70 kDa band precipitated from gL^+^ and gL^−^ lysates by heparin-agarose corresponds to gp70 [2].

The immune serum more strikingly detected the characteristic bands of virion gB-a minor 120 kDa full length form and its more abundant 65 kDa N-terminal and 55 kDa C-terminal cleavage products [18], [21]-whenever the 85 kDa band was present. The presence of gB was confirmed by immunoblotting the same precipitates with a gB-specific mAb (Fig. 3C). The precipitation of gB by heparin-agarose was initially puzzling, since we have previously shown that gB does not bind to GAGs [2]. Specifically, gB-N-Fc binding to cells is GAG-independent; heparin-agarose does not precipitate gB from Triton X-100 virion lysates; and the “heparin-binding” motif of gB is buried rather than accessible in its crystal structure [2], [19]-even allowing for gB conformation changes akin to those of the Vesicular stomatits virus glycoprotein G [22], it is hard to see how this motif could participate in GAG binding. However, gB precipitation from digitonin lysates by heparin-agarose was entirely consistent with gH/gL binding to GAGs, since in digitonin gB remains attached to gH/gL [6]. This is seen by gB being precipitated from gL^+^ and gL^−^ lysates by a gH-specific mAb and just from gL^+^ lysates by a gH/gL-specific mAb (Fig. 3B, 3C). The co-precipitated gB was more obvious than the precipitated gH/gL in Fig. 3B simply because the MuHV-4-immune rabbit serum, which recognizes gB [18], immunoblots gB better than it immunoblots gH.

### MAb blockade of GAG binding by gHL-Fc

**Figure 4:**
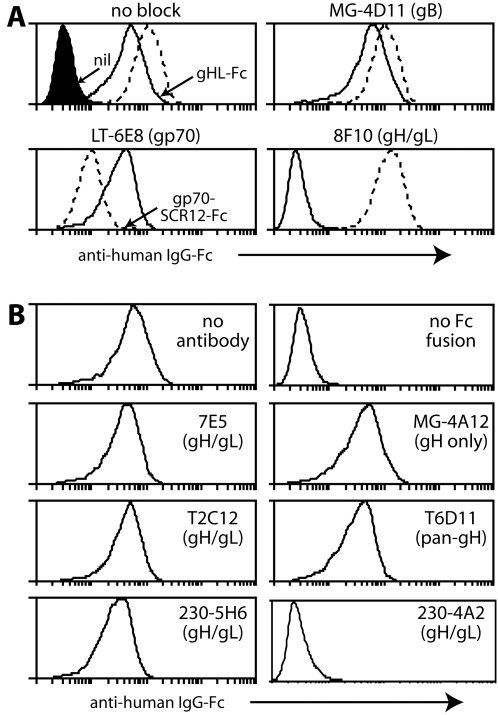
Inhibtion of Fc fusion protein binding by mAbs. A. Gp70-SCR12-Fc (dashed lines) = SCR domains 1+2 of gp70. gHL-Fc (solid lines)  = gH fused to C-terminal gL. Each Fc fusion protein was preincubated or not with mAbs as indicated and then used to stain BHK-21 cells. The filled histogram (nil) shows secondary antibody only staining. Similar data were obtained in 2 repeat experiments. B. Further gH-specific mAbs (see main text) were tested for their capacity to block gHL-Fc binding to BHK-21 cells.

Further support for a physiologically relevant interaction between gH/gL and GAGs came from antibody blocking (Fig. 4). In Fig. 4A, mAb LT-6E8 specifically inhibited gp70-Fc binding, mAb 8F10 specifically inhibited gHL-Fc binding and a gB-specific mAb inhibited neither. The gH/gL-specific mAbs 230-4A2 and 230-5B2 also completely blocked gHL-Fc binding. Several other gH/gL-specific mAbs had partial effects, but multiple IgM, IgG_2a_, IgG_1_ and IgG_2b_ control mAbs failed to block either Fc fusion protein (data not shown). Thus, the blocking was antigen-specific.

All our gH/gL-specific mAbs recognize conformational epitopes on MuHV-4-infected cells. Thus, their capacity to block GAG binding by gHL-Fc argued that this involves native gH/gL epitopes. The capacity of gH/gL-specific mAbs to block gHL-Fc binding did not particularly correlate with virion neutralization (Fig. 4B). For example, mAbs 7E5 and T2C12, which define the two major gH/gL neutralization epitopes [13], both failed to inhibit gHL-Fc binding. And while the gHL-Fc blocking mAbs 8F10, 230-4A2 and 230-5B2 all have neutralizing activity, it was less than that of 7E5 or T2C12 (data not shown). Indeed, gHL-Fc blocking does not necessarily explain the neutralization by 8F10, 230-4A2 or 230-5B2; an inhibition of membrane fusion is also possible. MAb 230-5H6 defines a gH/gL epitope that is normally protected by the gB N-terminus, but is a neutralization target when the gB N-terminus is deleted [20]. It failed to block gHL-Fc binding. Nor did gH-specific mAbs block binding-MG-4A12 provides a representative example-consistent with gH-Fc failing to bind GAGs (Fig. 1B).

### MAb blockade of cell binding by MuHV-4 virions

**Figure 5:**
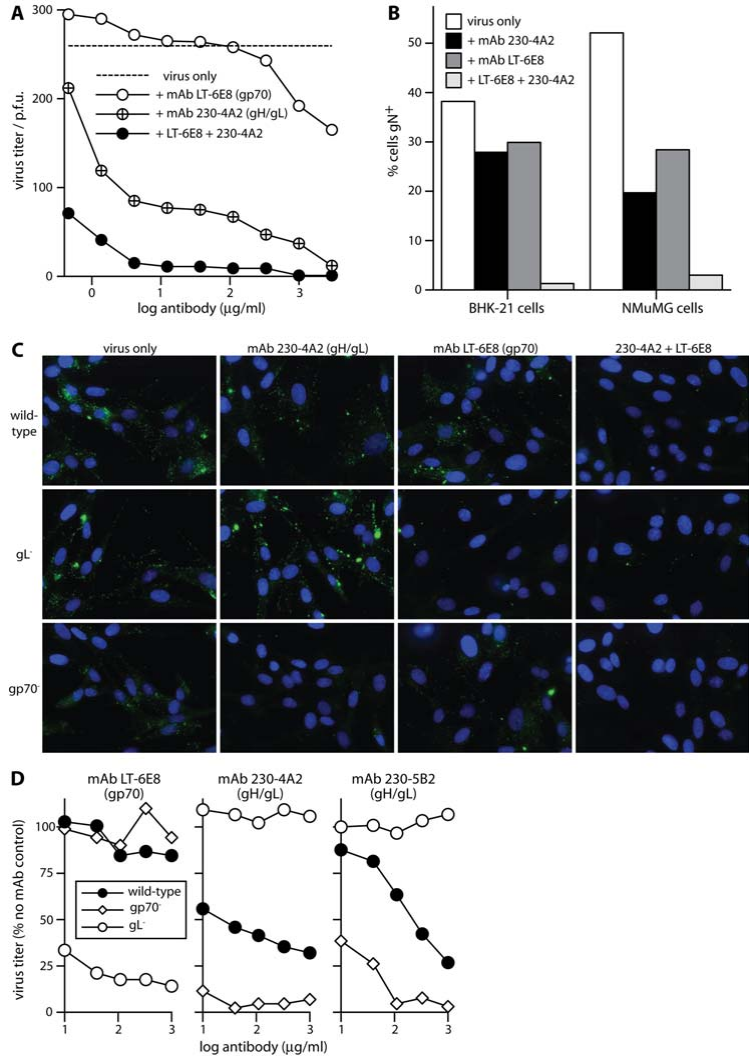
Inhibition of MuHV-4 infection by gp70-Fc and gHL-Fc blocking mAbs. A. Wild-type virions (200 p.f.u.) were incubated with mAbs as shown (2h, 37°C) then plaque-assayed on BHK-21 cells. We compared pairs of treatment arms by scoring (+ or -) for each antibody dilution whether the titer of a given treatment arm was more or less than the mean of both. We then performed an exact Chi-squared test on the comparisons. By this measure, mAb 230-4A2 but not mAb LT-6E8 gave significant neutralization, and 230-4A2 plus LT-6E8 was significantly better than 230-4A2 alone (p<0.0001). Equivalent data were obtained in 2 further experiments. B. Wild-type virions were incubated with mAbs (2 h, 37°C, 1 µg/1000 p.f.u.) then bound to either BHK-21 or NMuMG cells (2 h, 37°C, 3 p.f.u./cell). Bound virions were detected by washing, fixing and permeabilizing the cells, then staining them for gN with mAb 3F7. Secondary detection was with Alexa488-conjugated goat anti-mouse IgG pAb. This would have detected mAbs LT-6E8 and 230-4A2 even if the gN epitope were masked. The cells were scored simply as positive or negative on staining. Each bar shows the % positive of 10,000 cells, based on a gate where unexposed cells were 0% positive. The differences in binding between no mAb block/single mAb block, and between single mAb block/dual mAb block were highly significant (p<0.0001 by Student's t test). Equivalent data were obtained in 1 further experiment. C. Wild-type, gp70-deficient or gL-deficient virions (200 p.f.u.) were each incubated with mAbs (2h, 37°C, 1 µg/1000 p.f.u.), then with BHK-21 cells (2h, 37°C, 3 p.f.u./cell) as in B. The cells were then washed x3 in PBS, fixed and permeabilized, stained for gN with mAb 3F7 (green), counterstained with DAPI (blue) and examined microscopically for virion uptake. As in B, the anti-mouse secondary antibody would also have detected bound 230-4A2 or LT-6E8, so we could be sure no virions were missed because mAb binding had masked gN. D. Wild-type, gp70-deficient or gL-deficient virions (200 p.f.u.) were each incubated with mAbs as shown (2h, 37°C) then plaque assayed on BHK-21 cells. 230-4A2 and 230-5B2 both block gHL-Fc binding; LT-6E8 blocks gp70-Fc binding. Titers are plotted as % of p.f.u. without mAb for each virus. We compared treatment arms by an exact Chi-squared test of the pairwise comparisons at each antibody dilution, as in A. By this measure, mAb LT-6E8 neutralized the gL knockout but not the wild-type or gp70 knockout, and mAbs 230-4A2 and 230-5B2 neutralized the wild-type and gp70 knockout-the gp70 knockout significantly better than the wild-type-but not the gL knockout (p<0.004). The data are from 1 of 5 equivalent experiments.

The weak GAG binding of gp150 [2] seemed unlikely to compensate directly for a lack of gH/gL or gp70. But gp70 and gH/gL could potentially compensate for each-other, preventing antibodies specific for one or the other from blocking virion binding. This would explain why gp70 is a poor neutralization target and why gH/gL-specific neutralizing mAbs act mainly after binding [13]. Consistent with such an idea, synergistic neutralization was observed between mAbs LT-6E8 and 230-4A2 (Fig. 5A). LT-6E8 alone had little effect except at very high dose; 230-4A2 alone gave some neutralization, possibly by inhibiting infection post-binding; but LT-6E8 and 230-4A2 together were strongly neutralizing. We observed a similar synergistic blockade of virion binding (Fig. 5B, 5C): either mAb alone had much less effect than both combined.

The capacity of gp70 and gH/gL each to compensate for a loss of GAG binding by the other was confirmed with knockout viruses (Fig. 5D). gL-deficient MuHV-4 was much more potently neutralized than the wild-type by mAb LT-6E8; and gp70-deficient MuHV-4 was more potently neutralized than the wild-type by the gH/gL blocking mAbs 230-4A2 and 230-5B2. Thus, virions showed redundancy in GAG binding between gp70 and gH/gL.

### Neutralization directed against GAG binding in the absence of gp150

Gp150 is a major determinant of MuHV-4 GAG dependence, since gp150^−^ viruses are much less GAG-dependent than the wild-type [1]. The main deficit of gp150 knockouts is poor virion release, presumably because their GAG-independent virions are recaptured onto the GAG-deficient surfaces of infected cells [1]. This phenotype implies that gp150 normally covers a cell-binding epitope. The protected epitope is unlikely to be on gH/gL or gp70, since it binds a non-GAG ligand. Also, gL^−^gp150^−^ [6] and gp70^−^gp150^−^ mutants (data not shown) both retain the increased binding to GAG-deficient cells of the gp150^−^ mutant.

**Figure 6:**
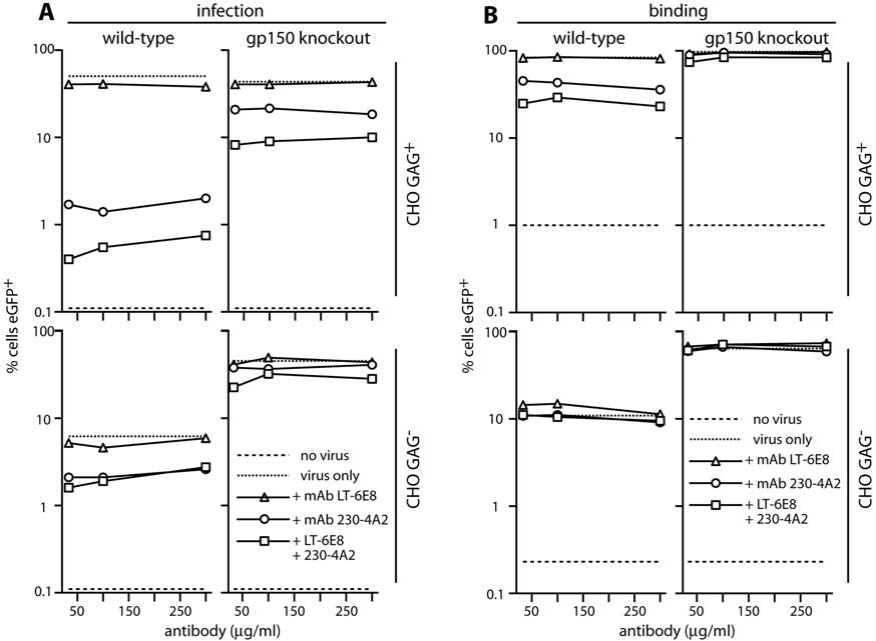
Gp150 deficiency alleviates an antibody-mediated block to GAG binding. A. EGFP-expressing wild-type or gp150 knockout virions were incubated with mAbs as shown, then used to infect GAG^+^ and GAG^−^ CHO cells. Infection was assayed 18h later by flow cytometry of viral eGFP expression, counting the %eGFP^+^ cells in a gate where uninfected cells (no virus) were <0.2% eGFP^+^. A log scale is used to encompass the large differences in infection between gp150^+^ and gp150^−^ viruses and between GAG^+^ and GAG^−^ CHO cells. Each point represents 10,000 cells. Equivalent data were obtained in a repeat experiment. B. gM-eGFP^+^ versions of the same viruses, which have eGFP^+^ virions, were incubated with GAG^+^ and GAG^−^ CHO cells (2h, 37°C) to allow binding/endocytosis. The cells were then washed x3 in PBS and assayed for eGFP uptake by flow cytometry. Each point represents 3,000 cells. Equivalent data were obtained in a repeat experiment. We compared viruses and cell types by taking the reduction in infectivity for each point of each of the 2 experiments (total n = 6). We then compared the reductions using Student's t test. This showed that mAb 230-4A2 and the double antibody treatment both neutralized wild-type MuHV-4 significantly better than the gp150 knockout, and that neutralization of the wild-type was significantly better with GAG^+^ CHO cells than with GAG^− ^(p<0.01).

We tested whether the gp150-regulated binding could compensate for a lack of gH/gL or gp70 binding by comparing the antibody-mediated blockade of gp150^+^ and gp150^−^ virions binding to GAG^+^ and GAG^−^ CHO cells (Fig. 6). For CHO GAG^+^ cells, antibody had less effect on either binding or infection when gp150 was lacking (compare the wild-type and knockout viruses). As expected, antibody was poorly effective against either virus infecting GAG^−^ CHO cells (where wild-type infectivity is anyway low). The modest reduction in infectivity with mAb 230-4A2 presumably reflected a post-binding block of membrane fusion. The complexity of virus/cell interactions must be borne in mind when interpreting these data: GAG^+^ and GAG^−^ CHO cells may differ in more than GAG expression; high virion avidity may allow gp70 and gH/gL to bind other polyanions in some settings; and gp150 deficiency may have knock-on effects on other virion glycoproteins. But the results argued strongly that gp70 and gH/gL between them provide the initial MuHV-4 cell attachment, and showed that GAG-independent binding can supplant gp70 and gH/gL only when gp150 is missing. GAG engagement by gH/gL and gp70 must therefore normally precede gp150 displacement.

### Heparin inhibition of MuHV-4 infection

**Figure 7:**
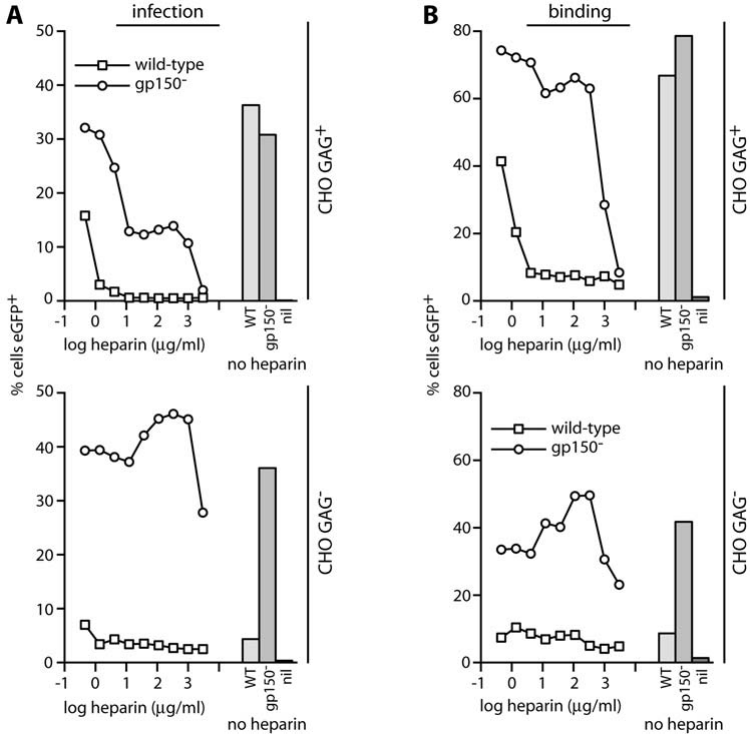
Comparison of MuHV-4 cell binding and infection in the presence of heparin. A. EGFP-expressing wild-type or gp150^−^ virions were incubated with different heparin concentrations, then used to infect GAG^+^ and GAG^−^ CHO cells. Infection was assayed 18h later by flow cytometry of viral eGFP expression, counting the %eGFP^+^ cells in a gate where uninfected cells (no virus) were <0.2% eGFP^+^. Each point represents 10,000 cells. The bars show infection levels without heparin. nil = no virus. Equivalent data were obtained in a repeat experiment. B. gM-eGFP^+^ versions of the same viruses were incubated with GAG^+^ and GAG^−^ CHO cells, with or without added heparin (2h, 37°C), then washed x3 in PBS and assayed for eGFP uptake by flow cytometry. Again, the bars show eGFP uptake without heparin, and nil = no virus. Equivalent data were obtained in a repeat experiment. The minor increases in gp150^−^ binding to and infection of GAG^−^ CHO cells at low heparin dose were quite variable between experiments; the reductions, particularly in wild-type MuHV-4 infection of and binding to GAG^+^ CHO cells, were not.

The dual role of GAGs in MuHV-4 entry-binding to gH/gL and gp70, and relieving the gp150 inhibition of GAG-independent binding-raised the question as to how soluble heparin inhibits infection. Heparin blocks wild-type MuHV-4 infection much better than that of a gp150 knockout [1]. But was the difference due to non-GAG binding rescuing gp150 knockouts, or to heparin blocking gp150 displacement rather than gH/gL/gp70 binding? To address this, we compared GAG^+^ and GAG^−^ CHO cell binding and infection by gp150^+^ and gp150^−^ MuHV-4 at different heparin concentrations (Fig. 7).

As before, each virus infected GAG^+^ CHO cells to much the same level, and the gp150 knockout infected GAG^−^ CHO cells much better than wild-type MuHV-4 did. Heparin also blocked wild-type MuHV-4 much better than the gp150 knockout for GAG^+^ CHO cell infection and binding, consistent with the antibody blockade differences in Fig. 6. As expected, heparin had little effect on either virus binding to or infecting GAG^−^ CHO cells. The key finding was that the binding and infection blocks for wild-type MuHV-4 on GAG^+^ CHO cells titrated out similarly. Thus, there was no evidence for heparin blocking a post-binding gp150 displacement better than the initial gp70/gH/gL cell binding. The data argued instead for a simple, heparin-mediated binding block from which gp150^−^ MuHV-4 could be rescued by GAG-independent binding. The non-availability of this ligand to gp150^+^ MuHV-4 fitted with GAG-dependent cell binding preceding gp150 displacement.

## Discussion

MuHV-4 infection is highly GAG-dependent. We previously identified gp150 as a key mediator of this dependence, in that disrupting gp150 makes infection GAG-independent [1], [2]. The present data refine this model by showing that MuHV-4 has 2 GAG-binding proteins-gp70 and gH/gL-which act upstream of gp150. Gp70 and gH/gL between them provide MuHV-4 with its vital first cell contact. MuHV-4 GAG dependence is consequently twofold: first gH/gL or gp70 must attach virions to cellular GAGs; then GAGs interact with gp150 to allow GAG-independent binding. We presume that gp150 displacement reveals an epitope on gB, since this is the only MuHV-4 glycoprotein known to have a non-GAG ligand. Direct evidence for a gB/gp150 interaction is lacking, but however gB is engaged, the different MuHV-4 GAG interactions are now seen to make a functionally coherent whole, combining efficient virion capture with down-stream binding initiated only at the cell surface.

Why does MuHV-4 have multiple GAG-binding proteins? The gp150/GAG interaction can be explained as triggering rather than adhesion, but this still leaves redundancy between gp70 and gH/gL. gL could have a particular role in gp150 displacement, since a gL deficiency somewhat reduces the virion gp150 content [6]. But gL^−^ virions show no qualitative difference in gp150 function, only a mild deficiency phenotype commensurate with their reduced gp150 content [6]. (Gp70 knockouts show no gp150 deficiency [2].) It seems more likely that gH/gL GAG binding allows for the effects of gp70 cleavage. Immunoblots indicate that BHK-21 cell-derived virions shed the GAG binding domain from at least 50% of their gp70 [2]. This may be important for efficient virion release, or for other gp70 functions such as complement evasion [7]. It may also be cell type-dependent [2]. If little gp70 were left uncleaved, GAG binding by gH/gL would be vital for attachment.

MuHV-4 is similar in its GAG-dependence not only to other herpesviruses [23] but also to unrelated viruses [24] and other, more complex intracellular pathogens [25]. GAGs evidently provide a useful general way to attach to and infect host cells. The ubiquity of GAG expression must confer on GAG-binding virions a general stickiness that helps to keep them cell-associated. Both virions and GAGs are inherently repetitive, so high avidity will tend to make their interaction irreversible. This emphasizes the general problem faced by virions passing between hosts, that they must combine efficient release from one epithelial surface with efficient penetration of the next. The predominant localization of GAGs to basolateral rather than apical epithelial surfaces [26] would allow MuHV-4 reactivating from a latently infected B cell to engage GAGs and infect epithelial cells basolaterally, before being released apically for transmission. Reduced GAG expression on infected cells [1] and shedding of the gp70 GAG binding domain [2] would compensate for any local breakdown of the epithelial barrier. The GAG shedding that accompanies inflammation [27] could also help. But the GAG-dependent virions would then have to reach basolateral GAGs to infect new hosts. Mucosal sampling pathways such as M cell transcytosis [28] may therefore be important for primary MuHV-4 infection: moving virions from the apical to the basolateral epithelial surface and initiating infection from underneath. Understanding herpesvirus transmission and how to prevent it clearly require that we learn more about virion uptake. The two-fold GAG-dependence of MuHV-4 implies that its incoming virions somehow reach the basolateral epithelial surface.

## Materials and Methods

### Viruses

All viruses were derived from a cloned MuHV-4 BAC [29]. Unless indicated, the loxP-flanked BAC cassette, which includes a human cytomegalovirus IE1 promoter-driven eGFP expression cassette, was removed by virus passage through NIH-3T3-CRE cells [30]. Gp70-deficient [29], gL-deficient [12] and gp150-deficient [1] MuHV-4 BAC mutants have been described, as have derivatives with eGFP-tagged glycoprotein M, which make fluorescent virions [21]. Virus stocks were grown and titered by plaque assay in BHK-21 cells [1]. Infected cells and supernatants were sonicated after harvesting, cell debris was pelleted by low-speed centrifugation (1000×*g*, 3 min), and virions were recovered from supernatants by high speed centrifugation (38000×*g*, 90 min).

### Cells

BHK-21 cells, NMuMG epithelial cells (American Type Culture Collection CRL-1636), CHO-K1 cells, the GAG-deficient CHO-745 mutant, NIH-3T3-CRE cells and 293T cells were all grown in Dulbecco's modified Eagle medium (Invitrogen, Paisley, U.K.) supplemented with 2 mM glutamine, 100 U/ml penicillin, 100 µg/ml streptomycin and 10% fetal calf serum (PAA laboratories, Linz, Austria). Cells were transfected using Fugene-6 (Roche Diagnostics Ltd., Lewes, U.K.).

### Plasmids

A human IgG_1_-Fc fusions of gp70 short consensus repeats 1+2 has been described [2], as has an equivalent fusion of the N-terminal half of gB [2]. The coding sequence for amino acid residues 1–450 of gp150 was PCR-amplified (Phusion DNA polymerase, New England Biolabs, Hitchin, U.K.) with 5′ *Xba*I-restricted and 3′ *Not*I-restricted primers and cloned into *Xba*I and *Not*I sites upstream of a human IgG_1_-Fc coding sequence as for gp70 and gB [2]. The gH extracellular domain was similarly fused to IgG_1_-Fc by PCR amplification of its extracellular domain (amino acid residues 1–703) with 5′ *Avr*II-restricted and 3′ *Not*I-restricted primers. gL missing most of its signal sequence was amplified with 5′ and 3′ *Not*I-restricted primers, and cloned in-frame into the *Not*I site between the gH extracellular domain and IgG_1_-Fc, as with a GPI-linked version of the same fusion [17]. The full-length gH extracellular domain was PCR-amplified with *Eco*RI and *Xho*I-restricted primers and cloned into the *Eco*RI/*Xho*I sites of pcDNA3 (Invitrogen Corporation). Expression plasmids for RACE-mapped gL, the gL ORF and gL-GPI have been described [13]. Fc fusion proteins were produced by transfecting the expression plasmids into 293T cells using. Supernatants were collected after 48h.

### Monoclonal antibodies

All mAbs described were generated from MuHV-4 carrier mice at least 6 months post-infection and selected for recognition of MuHV-4-infected BHK-21 cells without permeabilization. Thus, they define native epitopes on the virion or virus-infected cell surface. These mAbs were: 7E5, T2C12 [13], 230-5H6, 230-4A2, 8F10 (all anti-gH/gL), 8C1 (recognizes both gH and gH/gL) [13], MG-4A12 (recognizes gH rather than gH/gL) [12], LT-6E8 (anti-gp70), MG-4D11 (anti-gB), MG-10C11 (anti-gB) [21], and 3F7 (anti-gN) [31]. MAb 230-5H6 is IgG_1_; MG-10C11 is IgM; 8C1 is IgG_2b_; all the others are IgG_2a_.

### Immunoprecipitation and Immunoblotting

Virions were lysed on ice for 45 min in 1% digitonin, 50 mM TrisCl pH 7.4, 150 mM NaCl, 5 mM MgCl_2_ with Complete protease inhibitors (Roche Diagnostics). Insoluble debris was pelleted by centrifugation (13000×*g*, 15 min). The lysates were then incubated with either heparin-agarose beads (Sigma Chemical Co., Poole, U.K.) or mAbs plus protein A-sepharose. The beads were washed ×5 in 0.1% digitonin and heated (95°C, 5 min) in Laemmli's buffer. The released proteins were then resolved by SDS-PAGE. For total protein detection, gels were fixed in 10% acetic acid/50% methanol and stained with Coomassie R250. For immunoblotting, the proteins were transferred to PVDF membranes (Perbio Science, Tattenhall, U.K.). The membranes were blocked with 10% non-fat milk in PBS/0.1% Tween-20, then incubated with the gB-specific mAb MG-10C11 or with an MHV-68-immune rabbit serum [32]. Bound antibody was detected with horseradish peroxidase-conjugated rabbit anti-mouse IgG pAb (Dako Cytomation, Ely, U.K.) or horseradish peroxidase-conjugated donkey anti-rabbit IgG pAb (APBiotech, Little Chalfont, U.K.), followed by washing in PBS/0.1% Tween-20, development with ECL substrate (APBiotech) and exposure to X-ray film.

### Flow cytometry

Cells exposed to HCMV IE1 eGFP^+^ or gM-eGFP^+^ MuHV-4 were trypsinized and analyzed directly for green fluorescence. For protein binding, cells were incubated with human IgG-Fc fusion proteins or with MuHV-4 glycoprotein-specific mAbs (1h, 4°C), washed ×2 in PBS, and then incubated with fluorescein-conjugated rabbit anti-mouse IgG pAb (Dako Cytomation) or phycoerythrin-conjugated goat anti-human IgG-Fc pAb (Sigma Chemical Co.). For total rather than cell surface glycoprotein detection, the cells were fixed in 2% paraformaldehyde (30 min, 23°C) and permeabilized with 0.1% saponin before staining. All cells were washed ×2 in PBS before analysis on a FACS Calibur (BD Biosciences, Oxford, U.K.)

### Immunofluorescence

BHK-21 cells exposed to MuHV-4, washed with PBS to remove unbound virions, fixed in 4% paraformaldehyde (room temperature, 30 min), permeabilized with 0.1% Triton X-100, stained for gN with mAb 3F7 plus Alexa 488-conjugated goat anti-mouse IgG pAb, washed ×2, counterstained with DAPI and visualised with an Olympus microscope plus Retiga 4000R camera line (Qimaging, Burnaby, Canada).
